# Antitumor effects and molecular mechanisms of action of natural products in ovarian cancer

**DOI:** 10.3892/ol.2020.12001

**Published:** 2020-08-20

**Authors:** Yun-Bo Yan, Qing Tian, Ji-Fang Zhang, Ying Xiang

**Affiliations:** 1 Laboratory of Oncology, Center for Molecular Medicine, School of Basic Medicine, Health Science Center, Yangtze University, Jingzhou, Hubei 434023, P.R. China; 2 Department of Cell Biology and Genetics, School of Basic Medicine, Health Science Center, Yangtze University, Jingzhou, Hubei 434023, P.R. China

**Keywords:** ovarian cancer, natural products, molecular mechanisms, antitumor

## Abstract

Ovarian cancer is a common malignancy and the second leading cause of mortality among females with genital tract cancer. At present, postoperative platinum drugs and paclitaxel-based chemotherapy is the gold standard treatment for ovarian cancer. However, patients who receive this chemotherapy often develop cumulative toxic effects and are prone to chemotherapy resistance. Therefore, it is necessary to determine more effective treatment options that would be better tolerated by patients. Recent studies have reported the therapeutic effects of numerous natural products in patients with ovarian cancer. Notably, these natural ingredients do not induce adverse effects in healthy cells and tissues, suggesting that natural products may serve as a safe alternative treatment for ovarian cancer. The antitumor effects of natural products are attributed to suppression of cell proliferation and metastasis, stimulation of autophagy, improved chemotherapy sensitivity, and induction of apoptosis. The present review focused on the antitumor effects of several natural products, including curcumin, resveratrol, ginsenosides, (−)-epigallocatechin-3-gallate and quercetin, which are increasingly being investigated as therapeutic options in ovarian cancer, and discussed the molecular mechanisms involved in cell proliferation, apoptosis, autophagy, metastasis and sensitization.

## 1. Introduction

Ovarian cancer ranks third after cervical and uterine corpus cancer among gynecologic cancers and is the second leading cause of mortality worldwide among females with reproductive tract malignancies (1). Histopathologically, the majority of ovarian cancers are classified as epithelial ovarian cancer and include serous, mucinous, endometrioid, transitional and clear cell carcinomas (2). Epithelial ovarian cancer may metastasize through intracavitary implantation and/or the hematogenous and lymphatic routes. Intraperitoneal metastasis is the most common route of dissemination (3,4). Patients are usually asymptomatic in the early stages of the disease and ~70% of patients with ovarian cancer are diagnosed at an advanced stage (5). At present, surgery followed by platinum drugs and paclitaxel-based chemotherapy is the gold standard treatment to inhibit disease progression. However, patients who receive this chemotherapy often develop cumulative toxic effects (including nephrotoxicity) and are prone to chemotherapy resistance (6,7). To date, ovarian cancer is associated with a poor prognosis (8); the 5-year survival rate of patients with this malignancy remains significantly low to be satisfactory in clinic. Therefore, newer drugs and novel treatment strategies are warranted to improve the prognosis of patients with ovarian cancer.

Owing to the high toxicity associated with conventional antitumor drugs, plant-derived natural products are being investigated as alternative or adjuvant treatments for a variety of cancer types (9). Conventionally, natural products are considered nutritional products and auxiliary medicine. Recent studies have proven the antitumor effects of natural products and demonstrated that they may reduce chemotherapy-induced toxicity. These phytochemicals primarily include curcumin, resveratrol, ginsenoside, quercetin, berberine and (−)-epigallocatechin-3-gallate (EGCG) (10). This review discusses the antitumor effects of several natural products and the molecular mechanisms of action of these agents in patients with ovarian cancer, with regards to their roles in proliferation, apoptosis, autophagy, metastasis and sensitization. The antitumor effects of several natural products and the molecular mechanism in ovarian cancer are reviewed, which includes the aspects of proliferation, apoptosis, autophagy, metastasis and sensitization.

## 2. Curcumin

Turmeric, the root of the *Curcuma longa* plant, is widely used in Indian curries and South Asian dishes, and has been used as a traditional medicine for thousands of years in India and China (11). Curcumin and its two related curcuminoids (demethoxycurcumin and bisdemethoxycurcumin) constitute the main active ingredients of turmeric. Reportedly, curcumin and curcuminoids possess strong antitumor, antioxidant, and anti-inflammatory properties owing to their interactions with multiple molecular targets (12). The antitumor effect and mechanisms of action of curcumin in ovarian cancer are listed in Table I.

### 

#### Anti-proliferative and pro-apoptotic activity

Excessive proliferation and inadequate apoptosis are the two significant characteristics of tumors, including ovarian cancer. The phosphatidylinositol 3-kinase (PI3K)/protein kinase B (AKT) signaling pathway participates in multiple cellular process, and activation of this pathway increases cell proliferation, invasion, migration, survival and chemotherapy resistance in ovarian cancer (13). The B-cell lymphoma-2 (Bcl-2) family controls the release of cytochrome C from mitochondria into the cytosol, where it binds to apoptotic protease activating factor-1, promoting the activation of caspase-9 then caspase-3, which leads to the intrinsic apoptosis. The Bcl-2 family includes the pro-apoptotic proteins (including Bad and Bax) and anti-apoptotic proteins (including Bcl-2, and Bcl-xl) (14). Curcumin downregulated the expression of the Bcl-2, while upregulating the expression of Bax and caspase-3 by repressing the PI3K/AKT pathway, leading to cell cycle arrest in G2/M phase and increased apoptosis of ovarian cancer cells (15). Concordantly, Watson *et al* (16) reported that curcumin downregulated AKT phosphorylation, Bcl-2 and survivin, so that curcumin activated the extrinsic and intrinsic apoptotic pathways via activation of caspase-8, caspase-9 and caspase-3 (16). Signal transducer and activator of transcription-3 (STAT-3), is a transcription factor and signal transducer, and the phosphorylated STAT-3 promotes tumorigenesis by stimulating cell proliferation and preventing apoptosis (17). Saydmohammed *et al* (18), reported that curcumin inhibited STAT-3 phosphorylation, which suppressed ovarian cancer cell growth (18). Treatment with curcumin suppressed ovarian cell motility by inhibiting STAT-3 phosphorylation by increasing interleukin (IL)-6 and IL-8 secretion (19). In addition, Ca^2+^ homeostasis is necessary for cell survival, and sarcoplasmic/endoplasmic reticulum Ca^2+^ transporting ATPase (SERCA) regulates cellular Ca^2+^ flux from the cytosol to the endoplasmic reticulum (ER) for storage (20). Seo *et al* (21), reported that curcumin inhibited SERCA activity and then disrupted Ca^2+^ homeostasis in ovarian cancer cells. Subsequently, a high concentration of Ca^2+^ in the cytoplasm promoted cell apoptosis (21).

MiRNAs (miRs), a class of short non-coding RNAs, regulating gene expression post-transcriptionally via binding to the 3′-untranslated regions of target mRNAs, are essential in malignant phenotype and treatment response in ovarian cancer (22). Du *et al* (23), reported that treatment with dimethoxy-curcumin promoted the apoptosis and inhibited the proliferation of ovarian cancer cells through upregulating the levels of miR-551a. A luciferase assay confirmed that miR-551a targeted insulin receptor substrate 2 (23), which had been validated to serve an anti-apoptotic role (24). In addition, it was demonstrated that the combination of dihydroartemisinin and curcumin arrested the cell cycle and promoted the apoptosis of ovarian cancer cells by upregulation of miR-124 and downregulation of its target midkine (25), which is significantly overexpressed in various cancer types to promote tumorigenesis and progression (26). Furthermore, curcumin promoted the apoptosis and inhibited the proliferation of ovarian cancer cells by inducing the expression of miR-9 (27).

#### Induction of autophagy

In ovarian cancer, autophagy serves a dual role that it may serve as an adaptation to stress to avoid cell death in cancer progression, while excessive autophagy may lead to cell death. Increased autophagy is also associated with resistance to chemotherapy (28). Autophagy is a highly conserved process that involves the formation of autophagosomes that engulf cellular proteins and organelles, and delivers them to the lysosomes. The mammalian target of the rapamycin (mTOR) signaling pathway regulates cell proliferation, survival and autophagy. The p70 ribosomal S6 protein kinase (p70S6K) is a major effector of mTOR phosphorylation (29). Liu *et al* (30), reported that curcumin inhibited the AKT/mTOR/p70S6K pathway to induce apoptosis and protective autophagy in ovarian cancer SKOV3 and A2780 cells, and treatment with autophagy-specific inhibitors markedly enhanced curcumin-induced apoptosis (30). Qu *et al* (31), reported that B19, a novel monocarbonyl analogue of curcumin, induced autophagy and ER stress-mediated apoptosis in ovarian cancer cells, and inhibition of autophagy with 3-methyladenine increased ER stress-mediated apoptosis (31). ER stress is referred to as the accumulation of unfolded or misfolded proteins in the ER lumen due to physiological and pathological conditions, which interfered with ER homeostasis and proper protein folding (32).

#### Anti-metastatic activity

Ovarian cancer metastasizes to adjacent organs via direct extension or through the dissemination of cancer cells that detach from the primary tumor (3). A family of proteolytic enzymes, called matrix metalloproteinases (MMPs), promote the metastasis of ovarian cancer by remodeling the tumor extracellular matrix (33). Two recent studies reported that curcumin markedly downregulated CD44 (the cell surface receptor of hyaluronic acid), MMP-9 and Rab coupling protein-induced phosphorylation of focal adhesion kinase (FAK; associated with the stabilization of actin and microtubule filaments, and regulating cancer cell motility), and eventually inhibited the invasion of SKOV3 cells (34,35). In addition, one study revealed that bisdemethoxycurcumin inactivated the nuclear transcription factor-kappa B (NF-κB) pathway by inhibiting oxidative stress, thereby reducing the expression of metastasis-associated proteins, including MMP-2, MMP-9 and vascular cell adhesion molecule-1 (VCAM-1) in SKOV3 cells (36). The VCAM-1-integrin interaction was clarified to be involved in the regulation of ovarian cancer cell invasion and metastatic progression (37).

#### Sensitization

Resistance to chemotherapeutic agents is a major barrier to the effective treatment of advanced ovarian cancer. The molecular mechanisms associated with resistance to chemotherapy in ovarian cancer include increased activity of the drug efflux pump mediated by the multidrug resistance protein 1 (MDR-1, also known as P-gp), increased repair capacity of DNA damage, decreased drug intake, and decreased apoptosis, as well as drug-induced cell cycle arrest (38). Curcumin may increase the sensitivity of ovarian cancer cells to therapeutic drugs. Yallapu *et al* (39), reported that curcumin induced sensitization of cisplatin-resistant ovarian cancer cells (A2780CP) to cisplatin by increasing apoptosis. Curcumin treatment downregulated the expression of Mcl-1 and Bcl-xl, two anti-apoptotic proteins of the Bcl-2 family, and suppressed the activity of β-catenin, a transcription factor that promoted the expression of cell survival genes by interacting with the TCF transcription factor (39). Zhao *et al* (40), reported that co-administration of curcumin and paclitaxel exerted a good antitumor effect in multi-drug resistant ovarian cancer cells (SKOV3-TR30) and in ovarian tumor-bearing nude mice, since curcumin reversed the resistance to paclitaxel by inhibiting the drug efflux mediated by MDR-1 (40). Zhang *et al* (41) reported that curcumin may resume LncRNA MEG3 levels in extracellular vesicles from cisplatin-resistant ovarian cancer cells. Upregulation of MEG3 reduced the expression of miR-214 in cells and in extracellular vesicles, thereby reducing cisplatin resistance (41). MiR-214 was a well-known miRNA in drug resistance, which enhanced cell survival and induced cisplatin resistance in ovarian cancer cells by directly downregulating the expression of phosphatase and tensin homolog (PTEN), a negative regulatory molecule of the PI3K/AKT pathway (42).

## 3. Resveratrol

Resveratrol is a natural polyphenolic compound derived from plants, including grapes, peanuts and *Polygonum cuspidatum* (43). Resveratrol possesses anti-inflammatory and antitumor properties, and protects the heart, nerves and kidneys (44). The antitumor effect and mechanisms of action of resveratrol in ovarian cancer are listed in Table II.

### 

#### Anti-proliferative and pro-apoptotic activity

The Warburg effect describes that, under the condition of sufficient oxygen, tumor cells are active in aerobic glycolysis (45). Through aerobic glycolysis, a large number of metabolites may be generated and plentiful biosynthesis may occur, which may satisfy the rapid and unlimited growth of tumor cells (46). Suppression of the Warburg effect is an effective way to treat cancer (47). In the mouse model, resveratrol significantly reduced glucose uptake by tumor cells (48). In ovarian cancer cells, resveratrol inhibited the biosynthesis of hexosamine, and interrupted protein glycosylation through activating glycogen synthase kinase-3β (GSK3β), and eventually triggered ER stress-mediated apoptosis (49). GSK3β is a kinase that phosphorylated and inactivated glycogen synthase, the final enzyme in biosynthesis of glycogen, which is the main form of glucose storage (50). Tino *et al* (51) proved that the combination of resveratrol and acetyl resveratrol inhibited the growth and metabolism of ovarian cancer cells more efficiently, and this growth restriction was due to decreased NF-κB protein and nuclear localization, which was responsible for vascular endothelial growth factor (VEGF) secretion (51). Furthermore, resveratrol downregulated the phosphorylation of AKT and GSK3β in a dose-dependent manner in ovarian cancer cells, as well as decreasing the activity of extracellular signal-regulating kinase (ERK), which subsequently suppressed the expression of cyclin D1, which facilitated cyclin-dependent kinases CDK4 or CDK6 in promoting cell cycle progression (52).

#### Induction of autophagy

In ovarian cancer, a recent study reported that resveratrol promoted autophagy and subsequent apoptosis in ovarian cancer cells by triggering the production of reactive oxygen species (53). Furthermore, it also revealed that resveratrol induced autophagy through enhancing the expression of Atg5, a key molecule for the elongation of the autophagosome membrane, and promoting cleavage from microtubule-associated protein 1 light chain 3 (LC3)-I to LC3-II (53). LC3-II, located on the membrane of autophagosomes, is a specific marker protein for autophagic activity (54). Beclin-1 mediates the localization of autophagy-related proteins and regulates the formation and maturation of autophagosomes by interacting with various proteins. Beclin-1 may also interact with the anti-apoptotic proteins of the Bcl-2 family, exerting a crosstalk between apoptosis and autophagy (55). Zhong *et al* (56) demonstrated that resveratrol enhanced autophagy by promoting the expression of beclin-1 and LC3-II through inactivation of STAT-3, and significantly induced growth arrest and death of ovarian cancer cells (56). In addition, resveratrol promoted the expression of the tumor suppressor gene, aplasia Ras homologue member I (ARHI) (57), and inactivated the STAT-3 signal pathway in ovarian cancer cells. Subsequently, resveratrol induced cell apoptosis, increased autophagy activity, and induced growth arrest (58). Concordantly, Ferraresi *et al* (59) reported that resveratrol increased autophagy via upregulation of beclin-1 and LC3 through induced ARHI and inactivated STAT-3, thereby attenuating the metastasis induced by IL-6 in ovarian cancer cells (59). They also demonstrated that resveratrol inhibited mTOR complex 1 by repressing AKT and activating AMP-activated protein kinase (AMPK), which inhibited protein synthesis and cell growth, and induced autophagy. When the mTOR pathway was inhibited by resveratrol, induced autophagy favored the survival of cells in the context of insufficient nutrition, likely leading to a dormant state (60).

#### Anti-metastatic activity

Ovarian cancer is known to directly metastasize to the peritoneal surface of adjacent organs (3). A previous study demonstrated that resveratrol decreased the level of cellular α5β1 integrin and enhanced hyaluronic acid secretion to the extracellular matrix, which inhibited the adhesion of ovarian cancer cells to the intestinal wall and decreased metastasis (61). Under hypoxic conditions, resveratrol may decrease the binding between ovarian cancer cells and mesothelial cells by downregulating the expression of VEGF, thereby preventing the migration of ovarian cancer cells induced by lysophosphatidic acid (62). The high expression of VEGF was associated with metastasis in advanced ovarian cancer (63).

#### Sensitization

A previous study has reported that resveratrol induces autophagy and promotes apoptosis in ovarian cancer cells (64). Therefore, resveratrol may improve chemosensitivity and prevent tumorigenesis under conditions of autophagy inhibitors (64). Aberrant activation of NF-κB may protect cancer cells against the apoptosis induced by pharmacological drugs, contributing toward drug resistance. Nessa *et al* (65) suggested that resveratrol sensitized ovarian cancer cells to the apoptosis induced by platinum drugs via downregulating NF-κB (65). In addition, Engelke *et al* (66) reported that resveratrol reversed the resistance of ovarian cancer cells to cisplatin by modulating molecular targets, including the EGFR or VEGFR family of receptor tyrosine kinases (66).

## 4. Ginsenosides

Ginsenosides constitute the major pharmacologically active ingredient in ginseng, and possess antitumor and antioxidant properties. In addition to enhancing immunity (67). Several ginsenoside compounds, including Rb1, Rg1, RG1, Rh1 and Rd, have been identified, and the majority of these, particularly ginsenoside Rg3 and Rb1 exhibit significant antitumor activity (68). The effect and mechanisms of action of ginsenosides in ovarian cancer are list in Table III.

### 

#### Anti-proliferative and pro-apoptotic activity

The Warburg effect is essential for tumor growth and metabolism. Li *et al* (69) reported that ginsenoside 20(S)-Rg3 downregulated phospho-STAT-3 and two metabolic enzymes, hexokinase and pyruvate kinase, which inhibited the glycolysis of ovarian cancer cells, thereby inhibiting the Warburg effect and preventing tumor growth and metabolism (69). A previous study reported that 20(S)-Rg3 may block the inhibition of miR-324-5p by H19. Increased miR-324-5p by 20(S)-Rg3 treatment inhibited the activity of pyruvate kinase isozyme type M2, thereby inhibiting the Warburg effect (70). Furthermore, Lu *et al* (71) reported that ginsenoside 20(S)-Rg3 upregulated the expression of miR-603 in ovarian cancer cells by downregulating the DNA methylation mediated by DNA methyltransferase 3 alpha. Increased miR-603 directly targeted hexokinase-2 mRNA and decreased hexokinase-2 expression. Therefore, ginsenoside 20(S)-Rg3 inhibited the Warburg effect, which was primarily manifested as a decreased lactate production, glucose consumption and *in vitro* proliferation, and weakened cell invasion and migration (71).

#### Autophagy induction

The effect of ginsenosides on autophagy is controversial. Reportedly, ginsenoside 20(S)-Rg3 inhibited autophagic flux by suppression of late-stage autophagosome maturation or degradation, and eventually induced apoptosis in cervical cancer cells (72). However, in ovarian cancer, ginsenoside 20(S)-Rg3 enhanced autophagy by upregulating autophagy-related molecules, including LC3-II, Atg5 and Atg7, thereby inhibiting the invasion and metastasis of ovarian cancer cells (73).

#### Anti-metastatic activity

Hypoxia-inducible factor-1 (HIF-1), ubiquitous in human and mammalian cells, is stable only under hypoxic conditions, and the stabilization of HIF-1α mediates tumor cell invasion and metastasis (74). The epithelial-to-mesenchymal transition (EMT) usually occurs prior to ovarian cancer metastasis, which decreases cell-cell adhesion. Liu *et al* (75) reported that ginsenoside 20(S)-Rg3 decreased the expression of HIF-1α by stimulating the ubiquitin-proteasome pathway, and inhibited EMT process, which was essential for metastasis (75). They also revealed that ginsenoside 20(S)-Rg3 upregulated prolyl hydroxylase domain protein 1 to cause degradation of HIF-1α under the conditions of normal oxygen (76). Furthermore, ginsenoside Rb1 inhibited hypoxia-induced EMT by downregulating miR-25 in ovarian cancer cells, which abrogated the suppression of miR-25 on the expression of EP300 (a transcriptional activator of E-cadherin) and E-cadherin (an essential molecule for adhesion between epithelial cells), thereby leading to an anti-metastatic effect (77). E-cadherin is involved in anchoring epithelial cells to each other by binding to the actin microfilaments through α- and β-catenin in the cytoplasm (78).

#### Sensitization

Drug resistance is a major clinical challenge that interferes with successful cancer therapy. The membrane transporter MDR-1 is located on the lipid rafts of the plasma membrane, and increased MDR-1 activity is an important contributor to multidrug resistance. Yun *et al* (79) reported that ginsenoside Rp1 repressed MDR-1 activity by redistributing lipid rafts, which reversed resistance to antitumor drugs, including doxorubicin (79). EMT is involved in drug resistance, as well as in metastasis. Deng *et al* (80) reported that the metabolite compound k of ginsenoside Rb1 specifically inhibited cell growth by inhibiting the Wnt/β-catenin signaling pathway and EMT process, and decreased the resistance of ovarian cancer stem cells to cisplatin and paclitaxel (80).

## 5. Quercetin

Quercetin is a natural polyphenolic compound abundantly present in fruits and vegetables; notably, red onions contain the highest levels of quercetin (81). Quercetin has a variety of pharmacological actions, including antitumor, antioxidant and anti-inflammatory activity, in addition to lowering blood pressure and blood lipid levels. Quercetin-induced cytotoxicity is rarely observed in healthy cells (82). The antitumor effects and mechanisms of action of quercetin in ovarian cancer are listed in Table IV.

### 

#### Anti-proliferative, pro-apoptotic and anti-metastatic activity

Quercetin exerts antitumor effects *in vitro* and *in vivo* (83). It was reported that quercetin inhibited the expression of survivin protein, and maintained the cell cycle at the G0/G1 stage, thereby inhibiting the proliferation and promoting the apoptosis of ovarian cancer cells (84). In addition, quercetin upregulated the expression of miR-145, and then activated caspase-8, caspase-9 and caspase-3, which induced the apoptosis of ovarian cancer cells (85). In line with this, Teekaraman *et al* (86) reported that quercetin induced the intrinsic apoptosis in ovarian cancer cells. This study revealed that quercetin decreased the expression of anti-apoptotic proteins, Bcl-2 and Bcl-xl, while increasing the expression of the pro-apoptotic proteins, Bax and Bad, leading to the activation of caspase-9 and caspase-3 (86). In addition, Liu *et al* (87) reported that quercetin induced apoptosis and protective autophagy through ER stress, and the phospho-STAT-3/Bcl-2 signaling pathway was involved in this pharmacologic action (87). With regards to the role of quercetin in anti-metastasis, one study revealed that 3,4′,7-O-trimethylquercetin inhibited the invasion and metastasis of ovarian cancer cells by decreasing the expression of MMP-2 and urokinase plasminogen activator (uPA) (88). MMP-2 was reported as an early regulator of metastasis, and uPA was reported to promote metastasis in ovarian cancer cells (88). However, the role of quercetin in autophagy in ovarian cancer remains unknown.

#### Sensitization

Several studies have investigated the combined treatment of quercetin and chemotherapy or radiotherapy in ovarian cancer, due to the sensitization function of quercetin. Wang *et al* (89), reported that quercetin aglycone induced caspase-3 activation and poly-ADP-ribose polymerase (PARP; a DNA repair enzyme) deactivation, which caused cell apoptosis and promoted the sensitivity of ovarian cancer cells to cisplatin. Furthermore, quercetin inactivated the pro-survival mitogen-activated protein kinase (MAPK)-ERK signal pathway, downregulated cyclin D1 expression, and upregulated p21 expression, thereby arresting cell cycle progression (89). Yang *et al* (90) reported that quercetin increased the sensitivity of ovarian cancer cells to cisplatin by inducing ER stress. Furthermore, this study reported that quercetin significantly inhibited STAT-3 phosphorylation, and then downregulated Bcl-2 expression, attenuating the anti-apoptotic effect of Bcl-2 (90). In line with this, Gong *et al* (91) reported that quercetin enhanced the sensitivity to radiotherapy by the aggravation of DNA damage and ER stress through activating p53, which led to increased p21 and Bax expression, and decreased Bcl-2 expression (91). In addition, Yi *et al* (92) verified that quercetin increased the sensitivity of SKOV3 cells to tumor necrosis factor-related apoptosis inducing ligand (TRAIL)-mediated apoptosis by upregulating the transcription of death receptor 5 (DR5), the receptor of TRAIL (92). A high expression of DR5 was associated with a poor prognosis in patients with ovarian cancer (93).

## 6. Berberine

Berberine, an isoquinoline alkaloid, is the main bioactive ingredient of *Coptis chinensis*. Its pharmacological activity includes anti-inflammatory, antioxidant and antitumor effects (94). The antitumor effects and mechanisms of action of berberine in ovarian cancer are listed in Table V.

### 

#### Anti-proliferative and pro-apoptotic activity

A previous study revealed that berberine inhibited the proliferation of tumor cells, and induced apoptosis and cell cycle arrest (95). Berberine alone or combined with cisplatin may induce ovarian cancer cells to arrest at the G0/G1 phase and enhance the activity of cell death-associated proteins, including caspase-8 and caspase-3, thereby promoting apoptosis and necrosis (96). Prostaglandin E2 (PGE2) is a bioactive lipid that promotes cell proliferation and tumor growth (97). The chemotherapy drug VP16 promoted the synthesis of PGE_2_ by increasing the free arachidonic acid in ovarian cancer, which led to the proliferation of surrounding non-apoptotic cells and tumor repopulation. However, berberine inhibited the two key enzymes (calcium-independent phospholipase A2 and cyclooxygenase-2) of PEG_2_ synthesis in the tumor microenvironment, which resulted in decreased synthesis of PEG_2_ and inhibited the phosphorylation of FAK, which inhibited the chemotherapy-induced repopulation (98). Furthermore, berberine served an antitumor role in ovarian cancer by inhibiting the expression of the human ether a-go-go-related potassium channel (hERG1). The hERG1 protein was considered to be a key factor in tumorigenesis, and its high expression level in ovarian cancer cells may be downregulated by berberine treatment (99).

#### Autophagy induction and anti-metastatic activity

To date, the role of berberine in autophagy induction and the inhibition of ovarian cancer metastasis remains unclear. However, with regards to other cancer types, berberine was reported to induce autophagy and exhibited anti-metastatic action. Berberine was demonstrated to inhibit the MAPK/mTOR/p70S6K and Akt pathways in gastric cancer cells, thereby inducing cytostatic autophagy and cancer cell cycle arrest (100). Berberine may inhibit cell migration by downregulating matrix metalloproteinase-3 (MMP-3) in gastric carcinoma cells, thereby exhibiting anti-metastatic activity (101). In addition, berberine inhibited the metastasis of endometrial cells by downregulating the expression of cyclooxygenase-2 (102).

#### Sensitization

Marverti *et al* (103) reported the effect of berberine on sensitization in ovarian cancer. Berberine inhibited the growth of cisplatin-resistant ovarian cancer cells through suppressing the expression of dihydrofolate reductase and thymidylate synthase, two enzymes that are essential for DNA biosynthesis and thus important targets for chemotherapy (103). In another study, berberine increased the expression of programmed cell death-4 (PDCD4) by inhibiting the expression of miR-21, a known molecule associated with ovarian cancer cisplatin resistance, thereby increasing apoptosis and enhancing cisplatin sensitivity (104). PDCD4, an important tumor suppressor, was revealed to be associated with the malignant phenotype of ovarian cancer (105). In addition, overexpression of miR-93 was involved in cisplatin resistance in ovarian cancer. Berberine increased the sensitivity of ovarian cancer cells to cisplatin through inhibiting the expression of miR-93, thereby upregulating the expression of its target gene, PTEN (106). Hou *et al* (107), demonstrated that berberine induced apoptosis through increased DNA damage and suppressed homologous recombination, thereby increasing the sensitivity of ovarian cancer cells to PARP inhibitors (107).

#### 7. (−)-Epigallocatechin-3-gallate

EGCG, a major component of green tea polyphenols, exhibited marked anti-angiogenic, antioxidant, anti-inflammatory and antitumor effects, among other beneficial pharmacological actions. Bioinformatic analysis has demonstrated that EGCG may affect a variety of signaling proteins in the cell cycle, including Jun, NF-κB, Bcl-2 and MMPs, and also inhibits DNA replication (108). The antitumor effect and mechanisms of action of EGCG in ovarian cancer are listed in Table VI.

#### Effects on proliferation, apoptosis and autophagy

EGCG promoted the apoptosis of ovarian cancer cells by upregulating the levels of tumor suppressor gene, p53, and cell cycle inhibitor, p21WAF1. As a result, EGCG arrested ovarian cancer cells in the G1/S phase (109). Furthermore, EGCG induced the expression of p21 in cells, thereby promoting apoptosis and arresting these cells in the G2/M phase (110). EGCG downregulated the expression of aquaporin 5 and then downregulated NF-κB, which subsequently induced apoptosis, and inhibited the proliferation and metastasis of SKOV3 cells (111). However, the role of berberine in autophagy in ovarian cancer remains unclear. In hepatocellular carcinoma, EGCG directly interacted with LC3-I protein and promoted the synthesis of LC3-II through a series of reactions, thereby increasing the autophagy activity of HepG2 cells (112).

#### Anti-metastatic activity

Overexpression of c-Myb in ovarian cancer tissues often leads to a poor prognosis, since c-Myb activates NF-κB and the STAT-3 signaling pathway, which promotes tumor growth, invasion and chemotherapy resistance. Tian *et al* (113) evaluated numerous natural products and reported that EGCG significantly inhibited migration by downregulating the expression of c-Myb in ovarian cancer cells (113). Furthermore, EGCC inhibited the metastasis of ovarian cancer cells by inhibiting the phosphorylation of c-Jun and NF-κB, resulting in the decreased expression of VEGF and the secretion of MMP-2 and MMP-9. Consequently, the adhesion of cancer cells to extracellular matrix proteins was weakened, and the supply of nutrients required for cell proliferation was reduced (108).

#### Sensitization

Previous studies have revealed the effects of EGCG combined with conventional chemotherapeutic drugs in ovarian cancer. Chan *et al* (114) reported that EGCG increased the cytotoxicity of cisplatin and enhanced its efficacy by 3–6 fold. Specifically, EGCG increased the oxidative stress of cisplatin-induced ovarian cancer cells through the generation of more reactive oxygen species to induce cell apoptosis (114). Copper transporter 1 (CTR1) of tumor cells may increase the uptake of cisplatin. However, cisplatin treatment rapidly promotes CTR1 degradation, and decreased CTR1 is associated with cisplatin resistance. Wang *et al* (115,116) reported that EGCG maintained the concentration of CTR1 in ovarian cancer cells, thereby increasing the cisplatin sensitivity (115,116). Furthermore, in paclitaxel-resistant ovarian cancer cells, the combination of EGCG and sulforaphane treatment induced apoptosis and cell cycle arrest by damaging DNA and decreasing the expression of Bcl-2 and human telomerase reverse transcriptase (hTERT), the major catalytic subunit of telomerase, which was involved in cancer cell survival. The study suggested that EGCG may overcome paclitaxel resistance (117).

## 8. Conclusions and perspectives

Although conventional chemotherapy is known to produce positive initial effects in the majority of patients with ovarian cancer, cumulative toxicities and drug resistance often lead to the failure of conventional chemotherapy. Previous studies have revealed that numerous natural products are less toxic to healthy cells and inhibit tumor growth and progression to prevent malignant tumors. The present review summarizes the antitumor effects of natural products, primarily their role in the stimulation of autophagy, induction of apoptosis and cell cycle arrest, and the inhibition of cell proliferation and metastasis. Therefore, the administration of natural products (alone or in combination with other drugs) may be considered a useful treatment strategy in patients with ovarian cancer.

However, the mechanisms of action of natural products as therapeutic agents for ovarian cancer are relatively complex. The majority of previous studies have focused only on activity at the cellular level, and only few animal models have been developed. Further research is warranted to investigate the potential therapeutic efficacy of natural products in experimental animal models and randomized clinical trials. Accurate knowledge of the pharmacokinetic profile of each natural product is essential to evaluate the *in vivo* effects of the natural product.

The following issues require greater attention in future studies: i) The exact mechanisms that contribute toward the activity of natural products in ovarian cancer require further detailed investigation; ii) experimental animal model studies and randomized clinical trials should be performed to evaluate the therapeutic efficacy of natural products in ovarian cancer; iii) the effects of natural products combined with conventional chemotherapy, target therapy or immunotherapy need to be determined; and iv) novel methods should be developed to isolate and identify bioactive compounds from a variety of plants deemed suitable as anticancer agents.

## Figures and Tables

**Table I.** The antitumor effect and mechanisms of action of curcumin in ovarian cancer.

First author, year	Form of curcumin	Dose	Ovarian cancer model	Findings	(Refs.)
Yu *et al*, 2016	Curcumin	40 µM	Cell line (SKOV3)	Downregulated PIK3/AKT, increased caspase-3 and Bax, downregulated Bcl-2, promoted apoptosis, inhibited the proliferation, and induced G2/M cell cycle arrest.	(15)
Watson *et al*, 2010	Curcumin	Variable	Cell lines (HEY, OVCA429, OCC1, SKOV3)	Downregulated AKT phosphorylation and AKT protein, decreased the expression of Bcl-2 and survivin, and increased susceptibility to apoptosis.	(16)
Saydmohammed *et al*, 2010	Curcumin	Variable	Cell line (HOSE 642, OVCA 420 and OVCA 429)	Inhibited STAT-3 phosphorylation, inhibited the expression of IL-6 and SOCS-3, and repressed cell motility.	(18)
Seo *et al*, 2010	Curcumin	–	Cell lines (PA-1, OVCAR-3)	Suppressed STAT-3 phosphorylation, suppressed IL-6 and IL-8 secretion, and inhibited ovarian cell motility.	(19)
Seo *et al*, 2016	Curcumin	15 µM	Cell lines (MDAH2774, SKOV3, PA-1)	Inhibited SERCA activity, disrupted Ca^2+^ homeostasis, and promoted apoptosis.	(21)
Du *et al*, 2017	Dimethoxy-curcumin	Variable	Cell lines (ES2, HO8640, HO8640PM, SKOV3) and ovarian cancer tissues.	Upregulated miR-551a, promoted apoptosis and inhibited proliferation.	(23)
Zhao *et al*, 2017	Dihydro-artemisinin and curcumin	–	Cell line (SKOV3)	Upregulated miR-124, decreased midkine, induced cell cycle arrest, and promoted apoptosis.	(25)
Zhao *et al*, 2014	Curcumin	60 µM	Cell line (SKOV3)	Increased miR-9, decreased p-AKT and FOXO1, inhibited proliferation, and induced apoptosis.	(27)
Liu *et al*, 2019	Curcumin	Variable	Cell lines (SKOV3, A2780 and HO8910)	Decreased cell viability and induced apoptotic cell death.	(30)
Qu *et al*, 2013	B19	5, 10, 15 µM	Cell line (HO8910)	Activated ER stress and autophagy.	(31)
Choe *et al*, 2018; Lv *et al*, 2013	Curcumin	–	Cell lines (SKOV3, OVCAR-3 and PA-1)	Downregulated CD44, MMP-9 and Rab coupling protein-induced phosphorylation of FAK, and inhibited. invasion	(34,35)
Pei *et al*, 2016	Bisdemethoxycurcumin	15 µM	Cell line (SKOV3)	Suppressed oxidative stress, inactivated NF-κB, downregu lated the expression of MMP-2, MMP-9 and VCAM-1, and inhibited metastasis.	(36)
Yallapu *et al*, 2010	Curcumin	10 or 20 µM	Cell lines (A2780 and A2780CP)	Downregulated the expression of Bcl-xl and Mcl-1, suppressed β-catenin expression and transcriptional activity, increased apoptosis, and induced sensitization to cisplatin.	(39)
Zhao *et al*, 2019	Curcumin	Variable	Cell lines (SKOV3 and SKOV3-TR30) and *in vivo* (mice)	Upregulated p53, inhibited MDR-1, and reversed the resistance to paclitaxel.	(40)
Zhang *et al*, 2017	Curcumin	1 µM	Cell lines (OVCAR-3 and SKOV3)	Upregulated LncRNA MEG3, downregulated miR-214, and decreased cisplatin resistance.	(41)

PI3K, phosphatidylinositol 3-kinase; AKT, protein kinase B; Bcl-2, B-cell lymphoma-2; STAT-3, signal transducer and activator of transcription-3; SOCS-3, suppressor of cytokine signaling 3; IL, interleukin; SERCA, sarcoplasmic/endoplasmic reticulum Ca2^+^ transporting ATPase; miR, miRNA; p-AKT, phosphorylated AKT; FOXO1, forkhead box O1; ER, endoplasmic reticulum; NF-κB, nuclear transcription factor-kappa B; MMP, matrix metalloproteinase; FAK, focal adhesion kinase; VCAM-1, vascular cell adhesion molecule-1; MDR-1, multidrug resistance protein 1; LncRNA, long non-coding RNA.

**Table II.** The antitumor effects and mechanisms of action of resveratrol in ovarian cancer.

First author, year	Form of resveratrol	Dose	Ovarian cancer model	Findings	(Refs.)
Tan *et al*, 2016	Resveratrol	25, 50 µM	Cell lines (A2780 and SKOV3) and *in vivo* (mice)	Decreased the uptake of glucose, inhibited glycolytic response, and inhibited cell growth.	(48)
Gwak *et al*, 2016	Resveratrol	50 µM	Cell lines (PA-1, MDAH2774 and SKOV3)	Interrupted protein glycosylation, induced ER stress, and increased apoptosis.	(49)
Tino *et al*, 2016	Resveratrol and acetyl-resveratrol	30 µM	Cell lines (SKOV3 and OVCAR-5)	Decreased NF-κB protein and nuclear localization, decreased VEGF secretion, and decreased cell growth and metabolism.	(51)
Vergara *et al*, 2012	Resveratrol	>50 µM	Cell lines (OVCAR-3 and SKOV3)	Downregulated cyclin D1, AKT and GSK3β, decreased ERK phosphorylation, inhibited proliferation, and decreased drug resistance.	(52)
Lang *et al*, 2015	Resveratrol	100 µM	Cell lines (OVCAR-3 and Caov-3)	Induced ROS generation, induced Atg5 expression and promoted cleavage from LC3-I to LC3-II, and triggered autophagy and apoptotic cell death.	(53)
Zhong *et al*, 2016	Resveratrol	100 µM	Cell lines (Caov-3 and OVCAR-3)	Inhibited STAT-3 phosphorylation, upregulated beclin-1, promoted cleavage from LC3-I to LC3-II, and increased autophagy.	(56)
Zhong *et al*, 2018; Ferraresi *et al*, 2017	Resveratrol	100 µM	Cell lines (OVCAR-3, OC-Caov-3 and NIH-OVCAR-3)	Upregulated ARHI, inactivated STAT-3, increased autophagy and apoptosis, caused growth arrest, and attenu ated the metastasis induced by IL-6.	(58,59)
Ferraresi *et al*, 2017	Resveratrol	100 µM	Cell line (NIH-OVCAR-3)	Inhibited mTORC1 by inhibiting AKT and activating AMPK, inhibited protein synthesis and cell growth, and induced autophagy.	(60)
Mikula-Pietrasi *et al*, 2014	Resveratrol	Variable	Cell lines (A2780 and SKOV3)	Decreased α5β1 integrin, enhanced hyaluronic acid secretion, inhibited cell adhesion, and decreased metastasis.	(61)
Park *et al*, 2007	Resveratrol	Variable	Cell line (ARPE-19)	Inhibited HIF-1α and VEGF in a dose-dependent manner, blocked the PI3K/AKT/mTOR pathway, and prevented the migration.	(62)
Nessa *et al*, 2012	Resveratrol	Variable	Cell lines (A2780, A2780^cisR^ and A2780^ZD0473R^)	Decreased the resistance to the platinum drugs via downregulating NF-κB.	(65)
Engelke *et al*, 2016	Resveratrol	10 µM	Cell line (A2780)	Modulated the EGFR or VEGFR family of receptor tyrosine kinases, and prevented the development of cisplatin resistance.	(66)

ER, endoplasmic reticulum; NF-κB, nuclear transcription factor-kappa B; VEGF, vascular endothelial growth factor; AKT, protein kinase B; GSK3β, glycogen synthase kinase-3β; ERK, extracellular signal-regulating kinase; ROS, reactive oxygen species; LC3, microtubule-associated protein 1 light chain 3; STAT-3, signal transducer and activator of transcription-3; ARHI, aplasia Ras homologue member I; IL, interleukin; PI3K, phosphatidylinositol 3-kinase; mTOR, mammalian target of rapamycin; mTORC1, mammalian target of rapamycin complex 1; AMPK, AMP-activated protein kinase; HIF-1α, hypoxia-inducible factor-1α; EGFR, epidermal growth factor receptor; VEGFR, vascular endothelial growth factor receptor.

**Table III.** The antitumor effects and mechanisms of action of ginsenosides in ovarian cancer.

First author, year	Form of ginsenosides	Dose	Ovarian cancer model	Findings	(Refs.)
Li *et al*, 2015	20(S)-Rg3	80,160 µg/ml	Cell line (SKOV3)	Downregulated p-STAT, decreased metabolic enzymes in glycolysis, inhibited the Warburg effect, and prevented cancer growth and metabolism.	(69)
Zheng *et al*, 2018	20(S)-Rg3	40, 80 µg/ml	Cell lines (SKOV3 and A2780) and *in vivo* (BALB/C nude mice)	Blocked the competitive inhibition of H19 on miR-324-5p, increased the suppression of miR-324-5p on pyruvate kinase isozyme type M2, inhibit Warburg effect, and retarded cancer growth.	(70)
Lu *et al*, 2019	20(S)-Rg3	40, 80 µg/ml	Cell lines (SKOV3 and A2780) *In vivo* (BALB/C nude mice)	Upregulated miR-603, reduced hexokinase-2, inhibited the Warburg effect, and suppressed cell growth and invasion.	(71)
Zheng *et al*, 2017	20(S)-Rg3	80 µg/ml	Cell line (SKOV3)	Upregulated LC3-II, Atg5, and Atg7, enhanced autophagy, and inhibited metastasis and invasion.	(73)
Liu *et al*, 2014	20(S)-Rg3	5 mg/kg	Cell lines (SKOV3 and 3AO)	Activated ubiquitin-proteasome pathway, decreased HIF-1α, inhibited EMT, upregulated E-cadherin, and decreased the invasion and metastasis of cancer cells.	(75)
Liu *et al*, 2017	20(S)-Rg3	80,160 µg/ml	Cell lines (SKOV3 and 3AO)	Promoted HIF-1α ubiquitin proteasome degradation and inhibited the invasion and metastasis of ovarian cancer cells.	(76)
Liu *et al*, 2017	Rb1	160 µg/ml	Cell lines (SKOV3, 3AO)	Inhibited miR-25/EP300/E-cadherin pathway, and inhibited EMT.	(77)
Yun *et al*, 2013	Rp1, Rh2, Rg3	5,10 µM	Cell lines (OVCAR-8)	Redistributed MDR-1, attenuated MDR-1 expression, and induced drug sensitization.	(79)
Deng *et al*, 2017	Rb1, compound K	Variable; 50 mg/kg for compound K	Cell lines (SKOV3 and HEYA8 CSCs), and *in vivo* (mice)	Inhibited Wnt/β-catenin signaling pathway, inhibited EMT, and decreased chemoresistance.	(80)

p-STAT, phosphorylated signal transducer and activator of transcription; miR, miRNA; LC3, microtubule-associated protein 1 light chain 3; HIF-1α, hypoxia-inducible factor-1α; EMT, epithelial-to-mesenchymal transition; MDR-1, multidrug resistance protein 1.

**Table IV.** The antitumor effects and mechanisms of action of quercetin in ovarian cancer.

First author, year	Form of quercetin	Dose	Ovarian cancer model	Findings	(Refs.)
Ren *et al*, 2015	Quercetin	Variable	Cell line (SKOV-3)	Inhibited proliferation, induced apoptosis, decreased survival, and inhibited cell cycle progression.	(84)
Zhou *et al*, 2015	Quercetin	50 µM	Cell lines (SKOV3, A2780)	Upregulated miR-145 expression, activated caspase-3, −8 and −9, and induced apoptosis.	(85)
Teekaraman *et al*, 2019	Quercetin	Variable	Cell line (PK-1)	Decreased Bcl-2, Bcl-xL, increased Bid, Bax, Bad, cyto chrome C, increased caspase-3 and −9, and induced. apoptosis	(86)
Yi *et al*, 2014	Quercetin	2 mg/kg	Cell lines (SKOV-3, OVCAR-3, TOV-21G and HOSE)	Activated caspase-3 and DR5, downregulated cell survival protein, induced apoptosis, increased TRAIL sensitization, and inhibited tumor growth.	(92)
Yamauchi *et al*, 2017	3,4′,7-O-trimethylquercetin	–	Cell lines (CRL-1978, CRL-11731 and SK-OV-3)	Inhibited expression of uPA and MMP-2, and inhibited invasion and metastasis.	(88)
Liu *et al*, 2017;	Quercetin	80 mg/kg	Cell lines (SKOV-3, OVCAR-8)	Induced ER stress, induced apoptosis, suppressed STAT-3	(87,90)
Yang *et al*, 2015		twice a week	and *in vivo* (nude mice)	phosphorylation, downregulated Bcl-2, and induced autophagy.
Wang *et al*, 2015; Yang *et al*, 2015	Quercetin aglycone	–	Cell lines (SKOV-3, OVCAR-8)	Activated caspase-3, deactivated PARP, induced apop tosis, deactivated MAPK-ERK pathway, downregulated cyclin D1, upregulated p21, and increased sensitivity to cisplatin.	(89,90)
Gong *et al*, 2018	Quercetin	100 µM	Cell lines (OV2008 and A2780)	Activated p53, induced ER stress, increased p21 and Bax, decreased Bcl-2, induced the aggravation of DNA damage, and induced radio-sensitization.	(91)
Yi *et al*, 2014	Quercetin	Variable	Cell line (SKOV3)	Promoted TRAIL-mediated apoptosis via upregulating the transcription of DR5.	(92)

miR, miRNA; Bcl-2, B-cell lymphoma-2; DR5, death receptor 5; TRAIL, tumor necrosis factor-related apoptosis inducing ligand; uPA, urokinase plasminogen activator; MMP, matrix metalloproteinase; ER, endoplasmic reticulum; STAT-3, signal transducer and activator of transcription-3; PARP, poly ADP-ribose polymerase; AMPK, AMP-activated protein kinase; ERK, extracellular signal-regulating kinase.

**Table V.** The antitumor effects and mechanisms of action of berberine in ovarian cancer.

First author, year	Form of berberine	Dose	Ovarian cancer model	Findings	(Refs.)
Marverti *et al*, 2013	Berberine	5, 10 µM	Cell line (OV2008)	Interfered the expression of dihydrofolate reductase and thymidylate synthase, and reversed cisplatin resistance.	(103)
Liu *et al*, 2013	Berberine	10 µM	Cell lines (SKOV3 and OVCAR3)	Inhibited miR-21 expression, increased PDCD4 expression, and enhanced cisplatin sensitivity.	(104)
Chen *et al*, 2015	Berberine	10 µM	Cell line (A2780)	Increased cisplatin sensitivity via miR-93/PTEN/Akt pathway, and induced G0/G1cell cycle arrest.	(106)
Liu *et al*, 2019	Berberine	100 µM	Cell line (OVCAR3)	Induced necroptosis and apoptosis via upregulating caspase-3 and −8.	(96)
Zhao *et al*, 2017	Berberine	5 µM	Cell line (SKOV3)	Suppressed calcium-independent phospholipase A2 and cyclooxygenase-2, decreased PGE2 synthesis, inhibited FAK phosphorylation, and inhibited chemotherapy-induced repopulation.	(98)
Hou *et al*, 2017	Berberine	10 µM	Cell lines (A2780, HEY, SKOV3, HO8910, HO8910PM and OVCAR3)	Induced oxidative DNA damage, downregulated homologous recombination repair, and increased sensitivity to PARP inhibitors.	(107)

PDCD4, programmed cell death-4; miR, miRNA; PTEN, phosphatase and tensin homolog; AKT, protein kinase B; PGE2, prostaglandin E2; FAK, focal adhesion kinase.

**Table VI.** The antitumor effects and mechanisms of action of EGCG in ovarian cancer.

First author, year	Form of EGCG	Dose	Ovarian cancer model	Findings	(Refs.)
Huh *et al*, 2004	EGCG	Variable	Cell lines (SKOV-3, OVCAR-3 and PA-1)	Induced G1 or G1/S cell cycle arrest, and promoted apoptosis.	(109)
Chen *et al*, 2013	EGCG	–	Cell line (A2780)	Induced G2/M cell cycle arrest and apoptosis, and increased cisplatin sensitivity.	(110)
Yan *et al*, 2012	EGCG	40 µg/ml	Cell line (SKOV3)	Downregulated aquaporin 5 and NF-κB, induced apoptosis, and inhibited proliferation.	(111)
Tian *et al*, 2019	EGCG	Variable	Cell line (OVCAR3)	Inhibited c-Myb, downregulated NF-κB and STAT-3, activated p38 MAPK, decreased MMP-2, and inhibited proliferation and migration.	(113)
Wang *et al*, 2015; Wang *et al*, 2015	EGCG	20 mg/kg	Cell lines (OVCAR3 and SKOV3)	Induced CTR1 expression, and increased cisplatin sensitivity.	(115,116)
Chen *et al*, 2013	EGCG and sulforaphane	20, 10 µM	Cell line (SKOV3)	Downregulated hTERT and Bcl-2, promoted DNA damage response, induced apoptosis, and arrested cell cycle.	(117)

EGCG, (−)-Epigallocatechin-3-gallate; NF-κB, nuclear transcription factor-kappa B; STAT-3, signal transducer and activator of transcription-3; MAPK, mitogen-activated protein kinase; MMP, matrix metalloproteinase; CTR1, copper transporter 1; hTERT, human telomerase reverse transcriptase; Bcl-2, B-cell lymphoma-2.

## Data Availability

Not applicable.

## Abbreviations

EGCG: (−)-Epigallocatechin-3-gallate
PI3K: phosphatidylinositol 3-kinase
Akt: protein kinase B
Bcl-2: B-cell lymphoma-2
STAT-3: signal transducer and activator of transcription-3
IL: interleukin
SERCA: sarcoplasmic/endoplasmic reticulum Ca2^+^ transporting ATPase
miR: miRNA
LC3: microtubule-associated protein 1 light chain 3
mTOR: mammalian target of rapamycin
ER: endoplasmic reticulum
EMT: epithelial-to-mesenchymal transition
MMPs: matrix metalloproteinases
FAK: focal adhesion kinase
uPA: urokinase plasminogen activator
VCAM-1: vascular cell adhesion molecule-1
MDR-1: multidrug resistance protein 1
PTEN: phosphatase and tensin homolog
GSK3β: glycogen synthase kinase-3β
VEGF: vascular endothelial growth factor
ERK: extracellular signal-regulating kinase
ARHI: aplasia Ras homologue member I
AMPK: AMP-activated protein kinase
HIF-1α: hypoxia-inducible factor-1α
MAPK: mitogen-activated protein kinase
TRAIL: tumor necrosis factor-related apoptosis inducing ligand
PGE2: prostaglandin E2
hERG1: human ether a-go-go related potassium channel
PDCD4: programmed cell death-4
CTR1: copper transporter 1
hTERT: human telomerase reverse transcriptase
PARP: poly ADP-ribose polymerase
